# Small Intestine on a Chip Demonstrates Physiologic Mucus Secretion in the Presence of *Lacticaseibacillus rhamnosus* Biofilm

**DOI:** 10.1002/bit.28989

**Published:** 2025-04-08

**Authors:** Sanat Kumar Dash, Cláudia N. H. Marques, Gretchen J. Mahler

**Affiliations:** ^1^ Department of Biomedical Engineering Binghamton University Binghamton New York USA; ^2^ Department of Biological Sciences Binghamton University Binghamton New York USA; ^3^ Binghamton Biofilm Research Center Binghamton University Binghamton New York USA

**Keywords:** fluid shear stress, microbiota, MUC2, mucus, small‐intestine‐on‐a‐chip

## Abstract

The small intestine is an area of the digestive system difficult to access using current medical procedures, which prevents studies on the interactions between food, drugs, the small intestinal epithelium, and resident microbiota. Therefore, there is a need to develop novel microfluidic models that mimic the intestinal biological and mechanical environments. These models can be used for drug discovery and disease modeling and have the potential to reduce reliance on animal models. The goal of this study was to develop a small intestine on a chip with both enterocyte (Caco‐2) and goblet (HT29‐MTX) cells cocultured with *Lacticaseibacillus rhamnosus* biofilms, which is of one of several genera present in the small intestinal microbiota. *L. rhamnosus* was introduced following the establishment of the epithelial barrier. The shear stress within the device was kept in the lower physiological range (0.3 mPa) to enable biofilm development over the in vitro epithelium. The epithelial barrier differentiated after 5 days of dynamic culture with cell polarity and permeability similar to the human small intestine. The presence of biofilms did not alter the barrier's permeability in dynamic conditions. Under fluid flow, the complete model remained viable and functional for more than 5 days, while the static model remained functional for only 1 day. The presence of biofilm increased the secretion of acidic and neutral mucins by the epithelial barrier. Furthermore, the small intestine on a chip also showed increased MUC2 production, which is a dominant gel‐forming mucin in the small intestine. This model builds on previous publications as it establishes a stable environment that closely mimics in vivo conditions and can be used to study intestinal physiology, food‐intestinal interactions, and drug development.

## Graphical abstract

Dash and coworkers have fabricated a small intestine on a chip, which encapsulates the physiology and biofilm of the small intestinal epithelium. The dynamic system showed villus differentiation within 5 days of flow, and the mucus layer and biofilm showed small intestinal characteristics. With a cost‐effective and simple fabrication process, the platform is well‐suited for studying small intestinal physiology in both health and disease, as well as for oral drug screening and toxicology analysis.

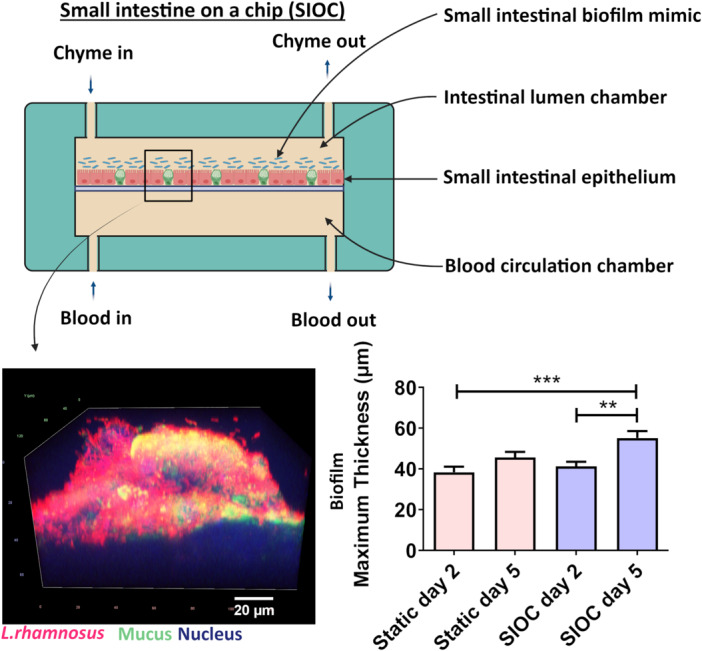

## 1 Introduction

The small intestine is the primary organ involved in food digestion and nutrient absorption. It is also a major route for drug delivery to the human body. The intestinal epithelium secretes digestive enzymes to assist the digestion process. The microbiota in the small intestinal lumen also helps break down food and supply essential nutrients (Smith and Morton 2010). Animal models for nutrient and drug absorption are time‐consuming and do not successfully replicate human biokinetic phenomena (Kulthong et al. 2020). Various 2D and 3D in vitro models have been developed to mimic small intestine physiology, but they too fail to successfully model the in vivo system. This is because they lack the mechanical environment of the intestinal epithelium which plays a large role in its function (Kim et al. 2012; Mercado‐Perez and Beyder 2022). The mechanical stresses acting on the epithelium are in the form of fluid shear and tension during peristalsis. Fluid shear stress alone has been known to initiate the differentiation of epithelial cells three times faster than static systems (Fois et al. 2021). Faster cellular polarization is achieved in organ‐on‐a‐chip models to reproduce in vivo physiology effectively (Kim et al. 2012). A dynamic in vitro model of the small intestine can help in the study of intestinal physiology in disease conditions and drug discovery, leading to time and cost savings in the drug discovery process, and can potentially serve as an alternate system to animal models (Ma et al. 2021; Mohs and Greig 2017; Monteduro et al. 2023). There is a consensus among experts that organs‐on‐a‐chip can increase the success rate and reduce R&D costs by 10%–26% (Carvalho et al. 2023).

The small intestinal epithelium consists of various cell types arranged in a simple columnar epithelium with a single layer of cells. The most prevalent cell type is the enterocyte, which constitutes approximately 80% of the cells within the small intestinal epithelium (De Santa Barbara et al. 2003). Enterocytes secrete digestive enzymes and absorb nutrients resulting from digestion. The second most common cell type is the goblet cell, which secretes and forms the mucus layer. The mucus layer rests on top of the intestinal epithelial layer facing the lumen. It acts as the first line of defense against agents such as the intestinal microbiota, mechanical forces, and enzymes (Duangnumsawang et al. 2021). The colon contains two mucus layers, one firmly adherent and another loosely adherent. Microbiota cannot cross the firmly adherent layer, which keeps the epithelium protected. In the small intestine the mucus layer is loosely attached, helping the interaction of microbiota with epithelial cells (Atuma et al. 2001; Corfield. 2001). The mucus is composed of mucin proteins that have a heavily glycosylated protein core. Mucin molecules bind to each other and form a net‐like structure. This net‐like structure holds water, which is the major component (≈ 95%) of the mucus membrane. Mucin sugars can bind viruses and pathogens, preventing their penetration through the mucus barrier (Carlson et al. 2018). The thickness of the mucus layer varies in different locations of the intestine. Small intestinal mucus has many antibacterial peptides and proteins to protect the epithelial layer from being invaded by microorganisms. There are two types of mucins found in the intestinal mucus layer, the membrane‐bound mucins and the secreted gel‐forming mucins. The four major gel‐forming mucins found in the GI tract are MUC2, MUC5AC, MUC5B, and MUC6. Among these, MUC2 forms the majority of intestinal mucins (Corfield 2001). Release of MUC2 proteins from the goblet cells occurs when microorganisms are present, as they help in the release of the enzyme meprin β that cleaves the anchor from goblet cells (Hansson 2019). MUC2 transcription is upregulated in the presence of bacteria such as *Pseudomonas aeruginosa* (Kim and Ho 2010). Probiotics such as *Lactobacillus spp*. enhance the mucus layer and glycocalyx to provide protection against pathogen invasion (Sherman et al. 2009). Based on their sialic acid composition, mucins can be divided into acidic or neutral mucins. Acidic mucins have more sialic acid groups and contain a higher percentage of amino acids threonine, proline, and glycine, whereas neutral mucins have less than 1% sialic acid and contain predominantly serine, alanine and aspartic acid (Wesley et al. 1985). Their percentage changes in disease conditions, as seen in cystic fibrosis patients where the percentage of neutral sugar increases and sialic acid decreases per total amount of carbohydrate (Carlson et al. 2018).

Gut and microbiota homeostasis is crucial in intestinal and overall health of the body. The microbiota is composed of various viruses, fungi, bacteria, and other microorganisms that cohabit with the mucus layer. The microbial population varies in different locations of the intestine. Within the small intestine, the bacterial population is dominated by Gram‐positive anaerobes. The predominant bacterial growth in the intestinal lumen occurs as biofilms, similar to those found in most environments (Buret and Allain 2023). When in biofilms, bacteria have different growth rates and gene expression patterns compared to their planktonic counterparts (Jandl et al. 2024). There have been some previous studies on intestinal epithelium and bacterial coculture (Han et al. 2023; Jalili‐Firoozinezhad et al. 2019) however, to our knowledge, there is no work focused on the biofilm development above the epithelial layer under the conditions used in this study. The bacterial population in the small intestine varies from 10^3^ colony‐forming units (CFUs) in the duodenum to 10^7^ CFUs in the ileum per gram of the luminal content (Malik et al. 2020; Sekirov et al. 2010). The microbiota promotes adaptive immunity against pathogens, and promotes the development of regulatory T cells, which prevent inappropriate responses to microbial antigens (Caruso et al. 2020). *Lacticaseibacillus rhamnosus*, a Gram‐positive, lactic acid bacterium, is generally found in fermented foods and is known for providing host barrier defense in the gut by secreting various factors such as short‐chain fatty acids (SCFA), hydrogen peroxide and bacteriocins (Dempsey and Corr 2022). Within in vitro systems, *L. rhamnosus* has been shown to improve transepithelial electrical resistance and tight junction formation in Caco‐2 cells by inhibition of NLRP3 inflammasome and autophagy (Feng et al. 2018). Genetic defects in humans change microbial population in the intestine leading to inflammation that could affect inflammatory bowel diseases (Cohen et al. 2019; Lavoie et al. 2019; Roda et al. 2020). Dysbiosis can also be found in other chronic diseases, including atherosclerosis, cirrhosis, hypertension, diabetes mellitus, and others (Sittipo et al. 2018). Diet can also change the microbial population in the intestine resulting in various metabolic diseases (Singh et al. 2017; Sittipo et al. 2018).

The primary objective of this study was to develop an in vitro small intestine on a chip (SIOC) that mimics small intestinal physiology. A mixture of two epithelial cell types, Caco‐2 and HT29‐MTX‐E12, replicated the enterocytes and goblet cells respectively, which are the predominant cell types in the intestinal epithelium. A biofilm of *L. rhamnosus* mimicked the small intestinal microbiota. The mechanical environment was simulated by the flow of media over the in vitro epithelium. Notably, the apical flow loop remained open to mimic the flow of chyme, whereas the basolateral flow was a closed loop which replicates the flow of blood. Lower shear stress and sufficient space on the apical chamber allowed biofilm mode of growth of the microbiota. The machining technology to fabricate our device was inexpensive and easily accessible. This device was used to successfully analyze the mucus layer secreted by the barrier and compare it with in vivo mucus secretion.

## 2 Material and Methods

### 2.1 Device Fabrication

The device was designed in Creo parametric and computational simulations were performed in Ansys Fluent to determine the shear stresses distribution on the channel walls. An incompressible working fluid was used in computations with laminar flow conditions. The density and viscosity of the fluid used was 1.009 g/cm^3^ and 0.93 mPa.s respectively, corresponding to the properties of cell culture media at 37°C (Poon 2022). The microfluidic device was comprised of two channels separated by a porous (0.4 μm) polycarbonate (PC) membrane (Sterlitech, Auburn, WA, USA). Devices were machined with a manual milling machine from PC sheets (thicknesses 0.25 in, 0.125 in and 0.04 in) and custom silicon gaskets (Mcmaster‐Carr, 0.04 in) were used to make a leakproof assembly.

### 2.2 Cell Culture

Human colon adenocarcinoma cell lines Caco‐2 and HT29‐MTX‐E12 were purchased from ATCC (Manassas, VA, USA) and Sigma‐Aldrich (Saint Louis, MO, USA) respectively. Both of the cell lines were cultured with Dulbecco's Modified Eagle high glucose medium (DMEM) without sodium pyruvate (Thermo Fisher Scientific, Waltham, MA, USA) supplemented with 10% heat‐inactivated fetal bovine serum (Thermo Fisher Scientific, Waltham, MA, USA) in a culture flask (Corning Life Sciences, Corning, NY). The culture medium was replaced 2–3 times a week and passaging was performed at 80% confluency using 0.25% Trypsin‐EDTA solution (Thermo Fisher Scientific, Waltham, MA, USA).

**Figure 1:**
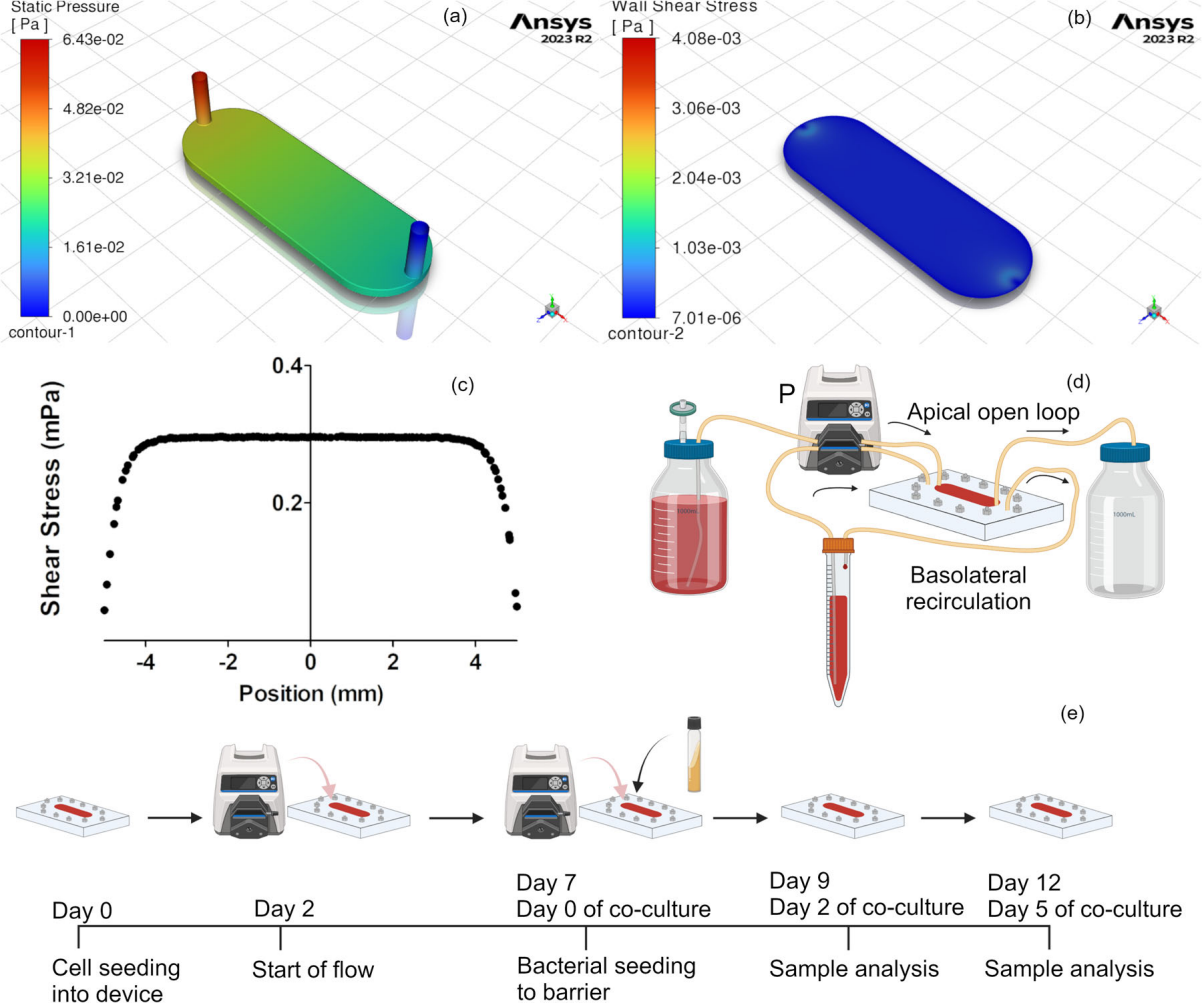
Design of the device and computational simulations (a) Pressure distribution in the apical chamber. (b) Shear stress distribution on the membrane wall. (c) Shear stress along with width of the membrane showing almost equal distribution. (d) Experimental setup showing recirculating flow in basolateral chamber and open loop flow in apical chamber (P‐peristaltic pump). (e) The experiment timeline shows a total of 12 days with 5 days of coculture with bacterial biofilm. (Created in BioRender. Marques, C. [2025] https://BioRender.com/a20z156J).

Caco‐2 cell lines between passage numbers 30–45 and HT29‐MTX‐E12 between passage numbers 55–70 were used for all experiments. Cells were trypsinized and seeded at a density of 200,000 cells/cm^2^ at a ratio of 3:1 (Caco‐2: HT29‐MTX‐E12) in all experimental conditions. Static cultures were grown in a 24 well Transwell plate (VWR, PA, USA) with 0.4 μm PC membrane. Cell culture surfaces were coated with rat tail collagen type I (BD Biosciences, San Jose, CA, USA) at a density of 8 µg/cm^2^ for 1 h at 37°C, 5% CO_2_ to facilitate cell attachment. For the SIOC, cells were allowed to adhere for 2 days in static conditions with a medium replacement every 24 h before the start of flow. A peristaltic pump (Watson‐Marlow 205S, MA, USA) with PharMed BPT tubing (0.51 mm internal diameter, VWR, USA) was used to initiate the flow. Borosilicate glass bottles (VWR, USA) and 15 mL centrifuge tubes (Corning, USA) were used as media reservoirs for the open loop circuit and closed loop circuit respectively. The flow continued for 5 days before bacteria were introduced to allow the barrier to differentiate and form tight junctions (Figure 1e). For static systems, medium changes were performed every other day. In figures, Day 0 refers to the cell status immediately before bacteria were added to the culture.

### 2.3 Eukaryotic Cell Viability Study

Cell viability was studied after 10 days of exposure to flow in the SIOC system. Samples were treated with a mixture of 2.5 µM Propidium Iodide (PI) (Thermo Fisher, MA, USA) and 3 µM Calcein AM (Thermo Fisher, MA, USA) and incubated for 30 min at 37°C and 5% CO2. Next, samples were imaged with a fluorescent microscope (Olympus BX43, Japan). Images were analyzed in ImageJ software using the intensity measurement function (Schneider et al. 2012).

### 2.4 Bacterial Cultures and Seeding on the Eukaryotic Cell Barrier

*Lacticaseibacillus rhamnosus* GG (ATCC 11775) was used as the intestinal microbiota mimic. In visualization experiments same species with a pDP195 plasmid with a mCherry transcriptional reporter, under spectinomycin selection (100 mg/L) (Cozy and Kearns 2010) was used. The strain originates from our laboratory and the plasmid was a kind gift from Dr. Laura Cook (the plasmid originated from Dr. Dan Kearns lab). The plasmid was introduced onto the *L. rhamnosus* via electroporation using previously described protocols (Schenk and Laddaga 1992). Overnight cultures were grown in brain heart infusion medium (BHI, Becton, Dickinson, Sparks, MD) supplemented with 0.05% l‐cysteine, 0.5% dextrose, 0.1% bacteriological agar (Complete BHI), and 100 mg/L of spectinomycin (when using the mCherry strain). The bacterial cultures were diluted in complete DMEM and seeded on top of the in vitro barrier at 5 × 10^3^ CFU/cm^2^ to mimic the upper small intestinal microbiota (García‐Rodríguez et al. 2020; Sekirov et al. 2010). The bacteria were allowed to attach to the eukaryotic cell layer for 1 h under static conditions after which the flow was resumed. After bacterial introduction, the apical chamber was fed with a culture medium diluted at a ratio of 1:3 with phosphate buffer saline (PBS) containing Ca (0.9 mM) and Mg (0.5 mM) and run in an open loop. The medium (complete DMEM) in the basolateral chamber continued to be circulated in a closed loop.

### 2.5 Permeability Assay

To analyze the robustness of the eukaryotic cell barrier in all experimental conditions, the apical chamber was supplemented with 50 μM Lucifer yellow (Thermo Fisher Scientific, Waltham, MA, USA). Lucifer yellow (LY) diffusion across the barrier was quantified by collecting 100 μL of media from the basolateral chamber at various time points and measuring its fluorescent intensity with a Synergy Neo2 plate reader (Agilent Technologies, Santa Clara, CA, USA). The following formula was used to calculate the apparent permeability of the system (Zhao et al. 2019).

$$P_{\mathrm{app}}=\left(\frac{\mathrm{dC}}{\mathrm{dt}}\right)\frac{{\;V}_{b}}{A\times C},$$

where,

$\left(\frac{\mathrm{dC}}{\mathrm{dt}}\right)$ is the rate of change of concentration of Lucifer yellow in the receiver or basolateral chamber.

$V_{b}$ is the Volume of the basolateral chamber.

A is the area through which transport occurs or the area of the membrane.

C is the initial concentration of Lucifer yellow in the apical or donor chamber.

### 2.6 Imaging of the SIOC and Static Culture

Laser scanning confocal microscopy (LSM 880, Carl Zeiss microscopy LLC) was used to visualize the microfluidic in vitro barrier. After the flow experiments the PC membranes were carefully removed from the device and fixed with 4% paraformaldehyde (PFA) (Thermo Fisher Scientific, Waltham, MA, USA) for 30 min at room temperature (RT). This was followed by permeabilizing the barrier with 0.2% Triton X‐100 for 15 min at RT. Unspecific binding of the primary antibody was prevented by incubating the sample with 1% bovine serum albumin (BSA) for 1 h at RT followed by overnight incubation with primary antibody (anti‐occludin (Invitrogen), aniti‐MUC2 (Invitrogen) or anti‐villin (ThermoFisher)). Next, secondary antibody (Alexa Fluor 568, ThermoFisher) was incubated for 2 h at RT. To stain with wheat germ agglutinin (WGA, Alexa Fluor 488 conjugate, Invitrogen) or phalloidin (Alexa Fluor 488, Thermo Fisher Scientific), samples were incubated with the specific stain for 2 h at RT. Finally, samples were counterstained with Hoechst for 30 min at RT. All the steps were followed by a wash step with 1× PBS. Samples were mounted on a slide with prolong gold antifade reagent (ThermoFisher) and imaged with the confocal microscope. Static samples were stained with the same protocols.

### 2.7 Mucus Layer Staining

To detect the mucins secreted by the in vitro barrier, samples were stained with Periodic Acid Schiff (PAS) (Sigma‐Aldrich, MO, USA) for all the mucins and Alcian Blue (AB) (Alfa Aesar, MA, USA) for acidic mucins (Limage et al. 2020). The membrane was carefully taken out from the device and washed with PBS. Samples were then fixed with 4% PFA for 30 min at RT. For acidic mucins, it was stained with AB in 3% acetic acid for 30 min at RT. For total mucin, samples were incubated with 0.5% periodic acid for 10 min at RT. Then, Schiff reagent was added to it for 15 min at RT. Finally, both the AB and PAS samples were washed with DI water until the water ran clear to remove any residual stain. Imaging was performed with an epifluorescent microscope (Olympus BX43, Tokyo, Japan) keeping constant imaging parameters for each image taken. Finally, images were analyzed with ImageJ software to quantify the intensity of the staining (supplementary data, ImageJ macro).

### 2.8 Biofilm Analysis

Samples were seeded with mCherry tagged *L. rhamnosus*. After the experiment, immediate fixation with 4% PFA (20 min at RT) was performed without disturbing the biofilm. Following this, staining of other components such as mucus (WGA) and nucleus (Hoescht) was carried out as described above. A wash with PBS was performed in between steps. Finally, samples were mounted on a microscopic slide and imaged with a Zeiss confocal microscope (LSM 880, Carl Zeiss microscopy LLC). Images were analyzed with the ImageJ plugin COMSTAT2 for properties including biomass, thickness, surface area to biovolume ratio, and surface roughness (Heydorn et al. 2000).

### 2.9 Statistical Analysis

All the experiments were run at least in triplicates with technical duplicates. Data were analyzed using GraphPad prism (GraphPad Software, San Diego California USA). Gaussian distribution of the data points was tested with D'Agostino and Pearson omnibus normality test. Significant differences between means were calculated with ANOVA followed by Tukey's or Bonferroni's posttest. The differences were considered significant when *p* ≤ 0.05.

## 3 Results

### 3.1 Device Design and Fabrication

The device dimensions were determined by considering the thickness of the mammalian cell layer and the thickness of the biofilm on top of it. The dimensions of the apical and basolateral chambers were kept the same to exert an equal pressure drop and shear stresses on the wall. This would lead to negligible crossflow across the membrane. Both the chambers had a thickness of 1 mm and width of 10 mm. Simulations were performed in Ansys Fluent to visualize the distribution of physical parameters such as pressure and shear stress. As shown in Figure 1a, the pressure drop across the device was negligible with an inlet flow rate of 30 µL/min. The same flow rate generated a roughly uniform shear stress of 0.3 mPa on the bottom of the chamber (Figure 1b,c). It shows the whole cell attachment area in the device is exposed to a similar mechanical environment. A lower magnitude of shear was used to prevent any biofilm disruption due to flow. This value of shear stress was also of the same order as seen in vivo and that was able to generate a differentiated in vitro epithelial barrier in earlier works (Delon et al. 2019; Lindner et al. 2021). Based on these parameters, the device was designed and fabricated using PC sheets sealed together with silicone gaskets (Supporting Information S1: Figure S1). The dimensions of the plate containing the channel and exploded view of components of the device are shown in Supporting Information S1: Figures S2 and S3, respectively. After assembly, to check if any instability in the pump or tubing could change the flow distribution in the device, both the chambers were fed with DI water and the weights of the collected liquid at the outlet were measured. The reading showed negligible maldistribution of flow across the membrane (Supporting Information S1: Figure S4). To ensure proper sealing between the chambers, the apical chamber was seeded with 10^6^ CFU/mL of *L. rhamnosus* and cultured in dynamic system. After 2 days, the media from the basolateral chamber was collected and drop‐plated to reveal the potential for *L. rhamnosus* to cross the barrier (Supporting Information S1: Figure S5), and these results showed no bacteria reached the basolateral chamber. The experimental setup was made where the apical chamber had an open loop flow while the basolateral chamber had a closed loop. This would mimic the one‐way flow of chyme through the intestine and recirculating blood flow respectively (Figure 1d).

### 3.2 In Vitro Epithelial Barrier Generation

**Figure 2:**
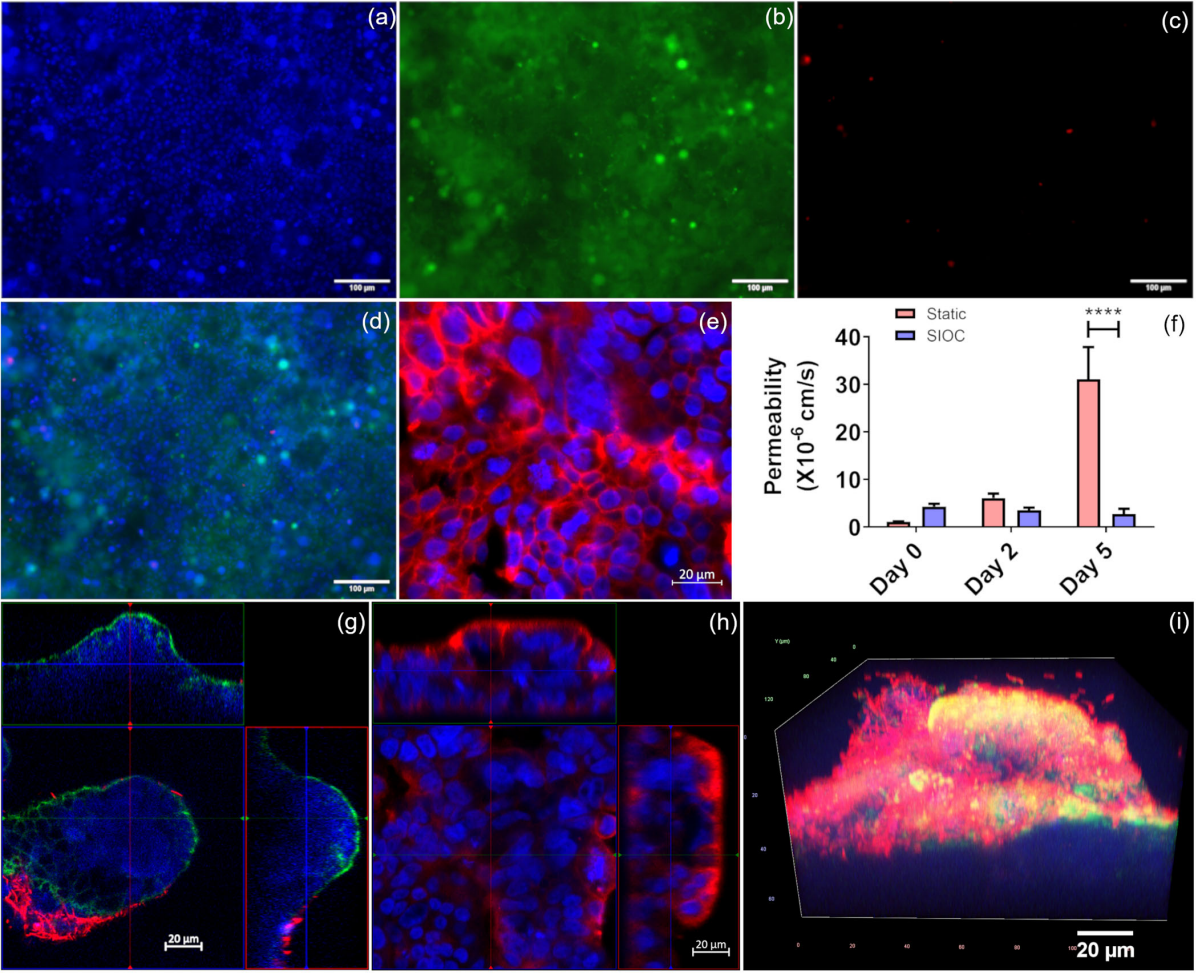
SIOC characterization. Images taken in the same location in the device for viability analysis (Day 10 after cell seeding) depicting (a) cell nucleus (Hoechst), (b) Calcein AM, showing live cell population (c) PI, showing dead cell population. (d) Merged image. Scale bar = 100 µm. (e) Tight junction formation (Day 7 after cell seeding) shown by anti‐occludin staining, red‐anti‐occludin, blue‐Hoechst, scale bar = 20 µm. (f) Lucifer yellow permeability comparison in the presence of *L. rhamnosus* in the static system, and SIOC (Day 0–5 of coculture with bacteria). (g) Epithelial differentiation shown by polarized epithelium with villi‐like structures along with *L. rhamnosus* at Day 2 of coculture with the bacteria (blue: mammalian cell DNA (Hoechst), green: F‐actin (Phalloidin), red: mCherry *L. rhamnosus*). (h) barrier differentiation shown by anti‐villin staining at Day 0 of coculture (red: villin, blue: Nucleus (Hoechst) scale bar 20 µm (i) 3D image showing barrier with thick *L. rhamnosus* biofilm and mucus (day 2 coculture). (blue: mammalian cell DNA (Hoechst), green: mucus (WGA), red: mCherry *L. rhamnosus*). Scale bar = 20 µm. Results are presented as mean ± SD of triplicate experiments. Significance was determined with two‐way ANOVA followed by Bonferroni posttest (*p < 0.05, **p < 0.01, ***p < 0.001).

The in vitro barrier remained viable after 10 days of flow in the device, showing long‐term viability of cells in the SIOC (Figure 2a–d). The images show a high percentage of live cells (green, Figure 2b) and a low percentage of dead cells (red, Figure 2c). The formation of tight junctions through anti‐occludin staining could be observed in the device after 5 days of culture with flow (Figure 2e). This shows the formation of a strong and intact epithelial barrier. To further validate the barrier function, the permeability of the barrier was determined with a Lucifer yellow transport assay. Figure 2f shows the permeability on the order of 10^−^^6 ^cm/s in the device, which is similar to values reported for differentiated in vitro epithelium in literature (Béduneau et al. 2014; Cheng et al. 2023; Ferraretto et al. 2018; Moreno‐Olivas et al. 2019). However, the role of fluid shear stress in maintaining the function of the SIOC could be observed in the device following *L. rhamnosus* introduction. As shown in Figure 2f the presence of *L. rhamnosus* significantly increased the permeability in the static system on day 5 of coculture, whereas it did not affect the permeability in the SIOC. There was also a significant increase in permeability observed in the static system on Day 2 of coculture as compared to Day 0 (Supporting Information S1: Figure S6). We also observed 3D projections in the barrier in the SIOC that mimic the intestinal villi. Such structures were either absent or very minimal in static systems. Figure 2g shows the formation of the 3D projection where filamentous actin is located at the border of the barrier showing its polarization. Localization of villin on the apical surface also confirms the differentiation of the cells to form the in vitro barrier (Figure 2h) (Gröne et al. 1986). A thick layer of *L. rhamnosus* biofilm on top of the villi was also observed during coculture as depicted in Figure 2i in red.

### 3.3 Mucus Analysis

**Figure 3:**
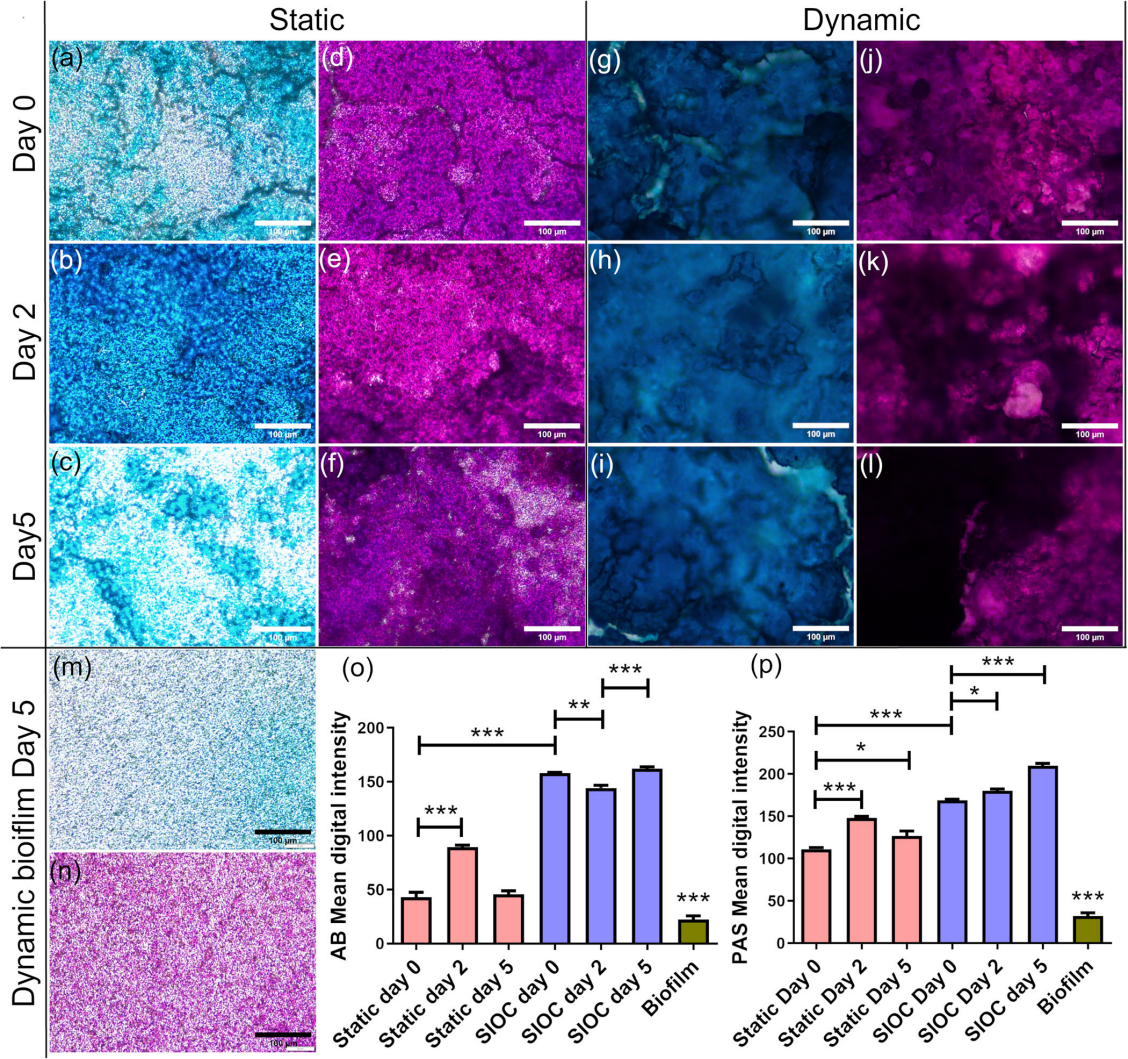
Mucus staining after static or SIOC culture. AB staining in the static system at (a) Day 0, (b) Day 2, (c) Day 5 of coculture. PAS staining in the static system at (d) Day 0, (e) Day 2, (f) Day 5 of coculture. AB staining in the SIOC at (g) Day 0, (h) Day 2, (i) Day 5 of coculture. PAS staining in the SIOC at (j) Day 0, (k) Day 2, (l) Day 5 of coculture. (m) AB and (n) PAS staining of 5 days culture of only the biofilm in the microfluidic device. Mean digital intensity quantification using ImageJ for (o) AB and (p) PAS. Scale bar = 100 µm. Representative images are presented. Results are presented as mean ± SD of triplicate experiments (at least 10 images from each sample). Significance was determined with one‐way ANOVA followed by Tukey's posttest (*p < 0.05, **p < 0.01, ***p < 0.001).

To study the mucus secreted by the in vitro barrier, samples were stained with AB, which stains the acidic mucins, and PAS, which stains both acidic and neutral mucins. Figure 3o,p shows that there was a significant increase in mucin secretion in the SIOC compared to the static system. In the static system, the AB staining increased on Day 2 of coculture but decreased significantly on Day 5 (Figure 3a–c). Whereas for the SIOC, the AB staining decreased slightly in Day 2 of coculture but was comparable to the initial condition on Day 5 (Figure 3g–i,o). An increasing trend was observed for PAS in the SIOC until Day 5 of coculture (Figure 3j–l,p). For the static system the total mucin staining peaked at Day 2 and decreased on Day 5 of coculture but was significantly higher than on Day 0 (Figure 3d–f,p). Interestingly, 2 days of flow with *L. rhamnosus* resulted in a significantly higher PAS staining but significantly less staining of AB (Figure 3o,p). This could indicate more neutral mucin secretion as compared to acidic mucins in this condition. To quantify the amount of stain uptake by the *L. rhamnosus* biofilm, the biofilms were cultured in the device with similar mechanical conditions. The images of biofilms alone revealed insignificant staining when compared to staining present in the in vitro barrier.

**Figure 4:**
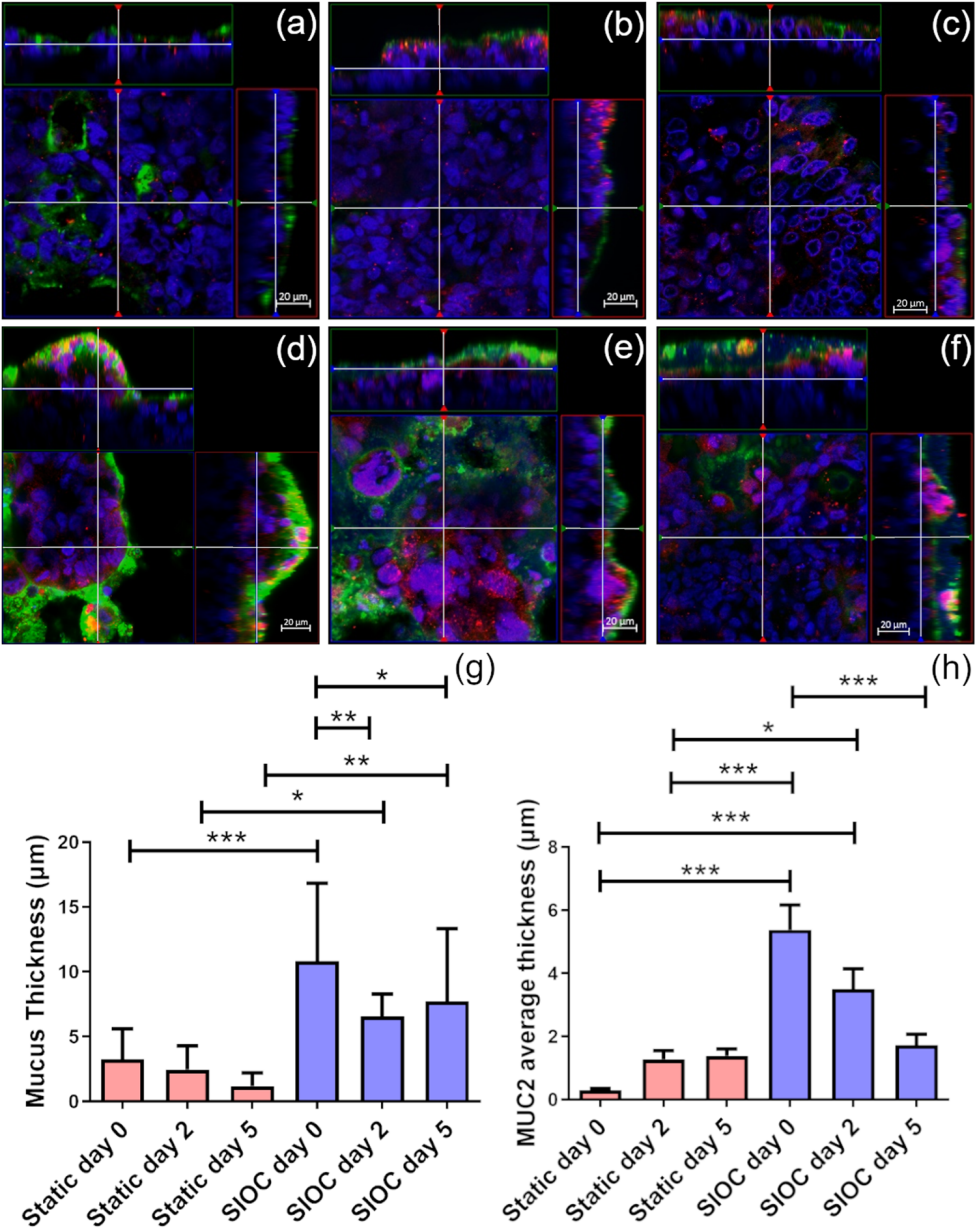
Confocal image analysis of mucus. Samples of the in vitro epithelium cocultured with *L. rhamnosus* were stained for mucus (WGA) and MUC2 and thickness was analyzed in ImageJ. (a–c) Static samples at Day 0, Day 2 and Day 5 of coculture respectively. (d–f) SIOC samples at Day 0, Day 2 and Day 5 of coculture respectively. (g) Mucus thickness analysis from WGA stain (μm). (h) MUC2 thickness analysis from MUC2 antibody stain (μm). Blue: nuclear material, Red: MUC2, Green: Mucus layer (WGA). Scale bar = 20 µm. Representative images are presented. Results are presented as mean±SD of triplicate experiments (68 images). Significance was determined with one‐way ANOVA followed by Tukey's posttest (*p < 0.05, **p < 0.01, ***p < 0.001).

Mucin was further analyzed with WGA staining and examining WGA‐stained mucus thickness from z‐stack confocal images (Supplementary data: Mucus/MUC2 thickness calculation). In Figure 4g, it can be observed that the overall mucus thickness in the SIOC is significantly higher than in the static system which was also confirmed by the AB/PAS staining images (Figure 3o,p). In the static system the thickness remains comparable throughout the 5 days of coculture, though their mean value reduces over time, whereas there is a significant reduction in mucus thickness in the SIOC. Comparing these findings with the ones for the AB/PAS staining, there is evidence that the adhered biofilm takes up some of the AB/PAS stain resulting in the increasing trend in PAS stain over time (Figure 3p). This could also indicate that the biofilm adhesion to the mammalian cell layer is more robust under the flow system compared to static system. Similar to the total mucus thickness, MUC2 thickness also reduced significantly in the SIOC on Day 5 of coculture. In the static system, the mucin thickness remained stable throughout the experiments though it was significantly lower than the SIOC (Figure 4g). HT29‐MTX cells do not generally secrete MUC2 under normal conditions (Lock et al. 2018). Interestingly, we observed significant MUC2 secretion in the dynamic in vitro model system. MUC2 was localized both in the mucus layer and in the cell layer (Figure 4). The average thickness of mucus layer in the device on Day 0 of coculture was 10.8 µm, which is close to the thickness of the firmly adherent mucus layer in the small intestine (Atuma et al. 2001; Corfield 2001).

### 3.4 Biofilm Development In the in Vitro System

**Figure 5:**
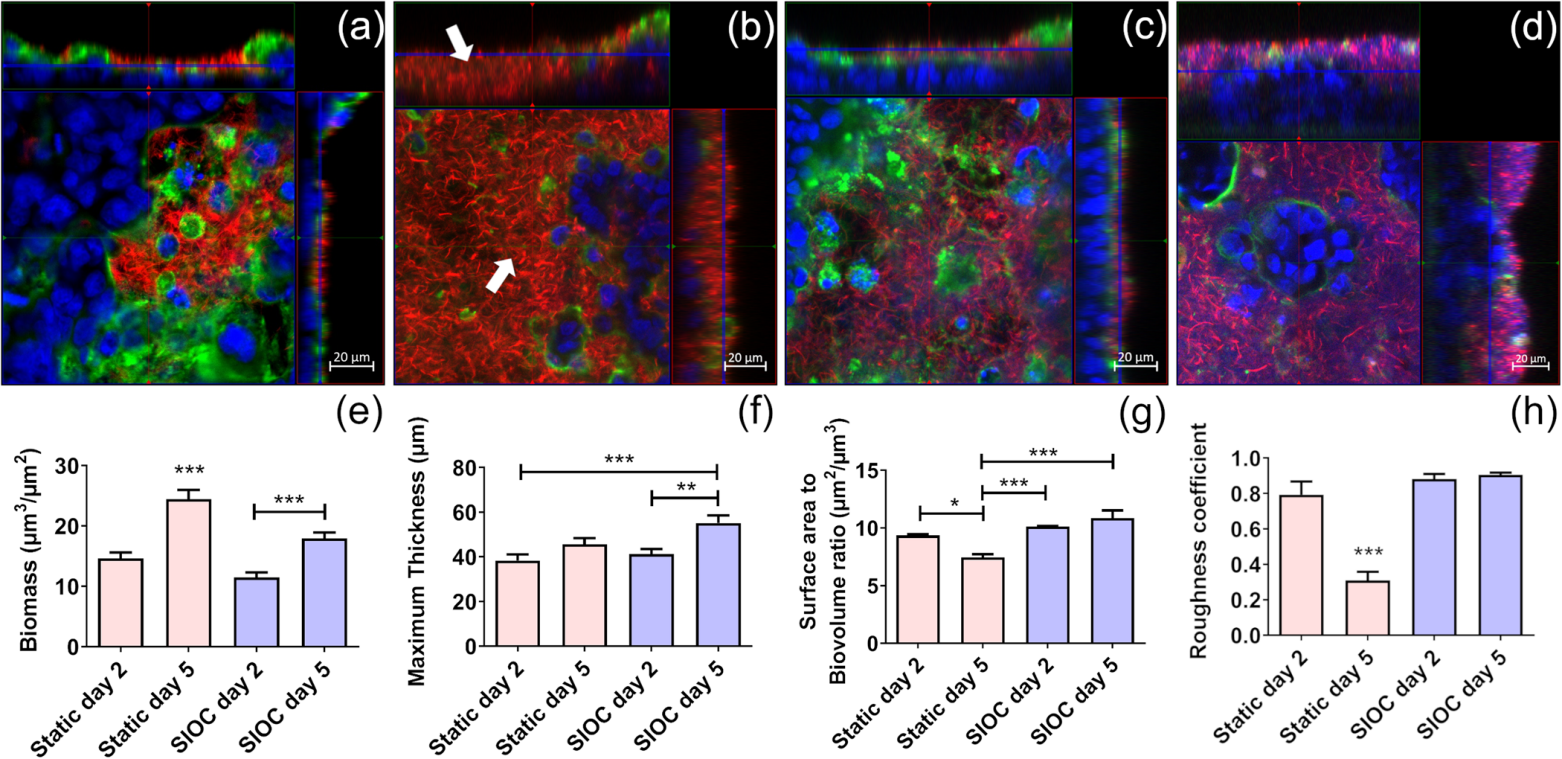
Analysis of biofilm formation on mammalian cells. Z‐stack images were acquired at Days 2 and 5 of coculture in both static system and SIOC. (a) static Day 2, (b) static Day 5, arrows indicating mCherry *L. rhamnosus* biofilm, (c) dynamic Day 2, (d) dynamic Day 5. At each time point various parameters of the biofilm were analyzed: (e) biomass (μm^3^/μm^2^), (f) maximum thickness from substratum (μm), (g) biofilm surface area to the total biofilm volume ratio (μm^2^/μm^3^), (h) Roughness coefficient for the biofilm. (red: mCherry *L. rhamnosus*; green: mucus; blue: DNA). Representative images are presented. Biofilm analysis was performed on 56 images from triplicate experiments. Significance was determined with one‐way ANOVA followed by Tukey's posttest (*p < 0.05, **p < 0.01, ***p < 0.001).

To visualize the biofilm formation on top of the mammalian cell layer a strain of *L. rhamnosus* constitutively expressing mCherry was used. Over time an increase in biofilm thickness was observed in both static system and SIOC (Figure 5a–d). In the static system it can be observed that the biofilm replaced the mammalian cell layer in several locations at Day 5 of coculture (Figure 5b). This correlates with the increase in lucifer yellow permeability in the in vitro epithelium in the static system (Figure 2f). This was not observed in the SIOC where the mammalian cell layer remained intact throughout the 5 days (Figure 5d) of coculture with the biofilms. The overall biomass of the biofilm was significantly higher at day 5 of coculture in the static system compared to the SIOC although it significantly increased from Day 2 to Day 5 of coculture in both static system and SIOC (Figure 5e), indicating the removal of excess biomass in flow. The maximum thickness of the biofilm from the substratum was highest on the Day 5 biofilm in the SIOC with no significant change in the static system from Day 2 to Day 5 (Figure 5f). The surface area to biovolume ratio and roughness coefficient was significantly decreased in static condition from Day 2 to Day 5, while in the SIOC these values were stable from Day 2 onwards (Figure 5g,h).

## 4 Discussion

Fluid flow has tremendous implications on gastrointestinal (GI) health. All cells in the gastrointestinal tract are sensitive to mechanical signals, which they are continuously exposed to (Mercado‐Perez and Beyder 2022). Mechanical activity in the gastrointestinal tract moves the luminal content in all directions, helping properly digest the luminal contents or the digesta. Overall, the digesta moves aborally due to the contractile gradient generated in the intestinal wall. This movement of digesta generates shear stress on the intestinal epithelial wall. The flow rate in the ileum and jejunum varies from 0.73 mL/min in a fasted state to 3 and 2.35 mL/min, respectively, in postprandial state (Lentle and Janssen 2008). The viscosity of the liquid phase of the digesta of dogs and pigs remains low and Newtonian when they maintain their natural diets (Dikeman et al. 2007). However, the viscosity of the whole digesta is non‐Newtonian and varies largely on shear rate and solid phase fraction (Lentle and Janssen 2008). Calculations taking the intestinal lumen as a circular tube show that the overall Reynold's number falls in the laminar range. Though the motion of the intestine can generate turbulence in the digesta, the presence of solid phases induces laminar mixing (Janssen et al. 2007). Such a mechanical environment results in a lower magnitude of shear stress on the intestinal wall. Previous works made use of shear stresses ranging from 0 to 3 mPa in their in vitro models (Delon et al. 2019; Fois et al. 2021; Kim et al. 2017; Lindner et al. 2021). They also made use of small dimensions in the microfluidic channels which could result in channel clogging due to mucus accumulation and inhibition of biofilm growth and 3D mammalian cell projections (Bakhtiari and Kähler 2024; Kim et al. 2006). Therefore, in this study, the behavior of the small intestinal epithelial barrier in low shear stresses of 0.3 mPa was studied. The channel dimensions used were significantly higher than earlier models, which facilitated the biofilm mode of growth of *L. rhamnosus*. Furthermore, device fabrication with photolithography is expensive and sophisticated and is not available to every lab. The conventional machining was used to fabricate the devices used in this study makes the fabrication inexpensive.

In previous microfluidic systems, fluid flow stimulated intestinal morphogenesis within 5 days (Shin and Kim 2022). Villus differentiation has been observed by mechanical forces including flow and cyclic strain (Kim et al. 2012). Shear stress increased the height of the cell monolayer as compared to static conditions and induced polarization (Fois et al. 2021; Kim et al. 2017). Flow has also previously been shown to increase mucus production (Kim et al. 2012). Organ‐on‐a‐chip models with the use of biopsy‐derived organoids have closely resembled the epithelium of duodenum (Kasendra et al. 2018). Sontheimer‐Phelps et al. differentiated goblet cells spontaneously from epithelial cells derived from patients in their device to get a mucus layer similar to the human colon (Sontheimer‐Phelps et al. 2020). These devices provide a method to study the role of mucus in various intestinal diseases. However, these are patient‐specific models and cannot be applied to the general population. Various cell lines have been used to generate standardized in vitro intestine‐on‐a‐chip models. For example, use of HT29‐MTX, a colon cancer cell line, resulted in in vivo‐like mucus secretion. It has been demonstrated that HT29‐MTX secretes a dense network of MUC5AC and MUC5B that generally mimics stomach and lung‐derived samples (Lindner et al. 2021). These cells also spontaneously differentiate towards villi‐like formations. Caco‐2, another colorectal adenocarcinoma cell line, is also extensively used in GI models. Caco‐2 can differentiate in culture and generate a polarized epithelium with tight junction formation. However, Caco‐2 cells do not secrete gel‐forming mucins.

A microbiota mimic, such as the commensal bacteria *L. rhamnosus*, has previously been demonstrated to improve barrier permeability and mucus production of an in vitro barrier from differentiated Caco‐2 cells (Kim et al. 2012). The presence of *L. rhamnosus* also decreased tissue damage when the pathogenic yeast *C. albicans* was introduced (Maurer et al. 2019). To obtain a physiologic microorganism population that mimics the gut microbiota, several studies have obtained and cultured fresh human stool specimens (Jalili‐Firoozinezhad et al. 2019). These models, however, lack reproducibility in the microbiota sample composition. Static models using Transwell inserts generally use planktonic cells instead of an adhered biofilm. Generally, systems containing a biofilm layer can sustain mammalian cell growth only for a short period before bacterial overgrowth leads to the loss of mammalian cell function and death (Jalili‐Firoozinezhad et al. 2019).

In the current device, similar observations were found in the dynamic condition within 5 days, with tight junction formation and villi‐like structure formation with a well‐developed mucus layer (Figure 2g,i) that was close to the thickness of the mucus layer in duodenum (Corfield 2001). The thickness remained significantly higher than static system after 5 days of coculture with *L. rhamnosus* (Figure 4g). Furthermore, 2 days of coculture of the in vitro epithelium with *L. rhamnosus* increased neutral mucin production but decreased acidic mucin production in the SIOC (Figure 3o,p). These data can be correlated with the healthy in vivo small intestinal conditions where there is a greater percentage of neutral mucin than acidic mucins (Corfield. 2001; Wesley et al. 1985). The SIOC also produced more MUC2, the predominant mucin in the small intestine compared to static system. The production of MUC2 was enabled by coculturing of Caco‐2 with HT29‐MTX (Lock et al. 2018). The total mucus layer was thicker than the MUC2 thickness (Figure 4g,h). The epithelial cells secrete other types of mucins such as MUC5AC and MUC5B, which could be the reason for the discrepancy. MUC2 could also get washed out during the immunocytochemistry process as it is a secretory mucin. There was a reduction in mucus layer thickness over time for both the total mucus thickness and MUC2 thickness in the SIOC. This overall decrease may be due to enzymatic degradation by the biofilm (Atuma et al. 2001). Microbiota thrive on specific O‐glycans and through this mechanism mucins control the bacterial population in the intestine (Luis and Hansson 2023). The surface‐to‐biovolume ratio of the *L. rhamnosus* biofilm significantly increased in dynamic conditions indicating a decreased nutrient supply to the interior of the biofilm microcolonies (Heydorn et al. 2000). In a biofilm, the bacterial population on the surface of the biofilm is exposed to more nutrients, whereas bacteria within the biofilm and at the bottom of the biofilm receive fewer nutrients and are metabolically less active and likely smaller in size (Anwar et al. 1992). As digesta moves through the GI tract, its nutrient concentration gradually decreases. Hence, low nutrient conditions for biofilms are essential to mimic small intestinal microbiota. The roughness coefficient of the biofilm remained higher in the SIOC which enabled them to survive in shear stress by preventing detachment (Shen et al. 2015). Protection against shear stress is crucial for biofilm survival in vivo (Jandl et al. 2024). Immune cells in Peyer's patches and the lamina propria play a large role in the adaptive and innate immunity of small intestine (Kobayashi et al. 2019). They are also involved in autoimmune diseases such as Crohn's disease. In the future, inclusion of immune cells to the SIOC would enable an on‐chip immune response. For an improved mimic of the small intestinal microbiota, a biofilm composed of multiple commensal bacteria could also be developed in the future. Overall, the SIOC demonstrates an improved system for mimicking small intestinal physiology.

## 5 Conclusion

In this study we developed a small intestine on a chip that enhances the currently available in vitro models. The device successfully mimics the dominant cells and flow parameters of the intestinal epithelium model. It incorporates *L. rhamnosus*, a commensal and probiotic microorganism, in a biofilm mode of growth, while also promoting 3D mammalian cell projections with a mucus layer thickness close to the one present in the duodenum. The dynamic steady conditions can be maintained for longer than 5 days. As such, this small intestine on a chip provides an advanced platform for intestinal physiology study with a straightforward and economical fabrication process enabling the study of oral drug screening and toxicology analysis including the role of the microbiota.

## Conflicts of Interest

The authors declare no conflicts of interest.

## Supporting information

Supplementary data revised.

## Data Availability

The data that support the findings of this study are available from the corresponding author upon reasonable request.
