# Supplementary material for: Small Intestine on a Chip Demonstrates Physiologic Mucus Secretion in the Presence of Lacticaseibacillus rhamnosus Biofilm
Source: Biotechnol Bioeng. 2025 Apr 8;122(7):1816–27. doi: 10.1002/bit.28989 (PMC12152466; doi:10.1002/bit.28989)
Supplement: Supplementary file 1 — Supplementary data revised. [file BIT-122-1816-s001.docx]

**Supplementary Material**

**Small Intestine on a Chip Demonstrates Physiologic Mucus Secretion in the Presence of *Lacticaseibacillus rhamnosus* Biofilm**

Sanat Kumar Dash^a,b,c^, Cláudia N. H. Marques^b,c^, Gretchen J. Mahler^a,c,*^

^a^Department of Biomedical Engineering, Binghamton University, Binghamton, NY, USA

^b^Department of Biological Sciences, Binghamton University, Binghamton, NY. USA

^c^Binghamton Biofilm Research Centre, Binghamton University, Binghamton, NY, USA

*Corresponding author

**1. Supplementary Figures**


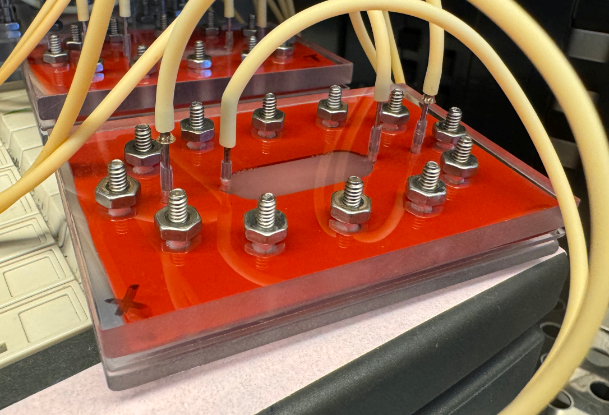


Figure S1: Fully assembled device with tubing


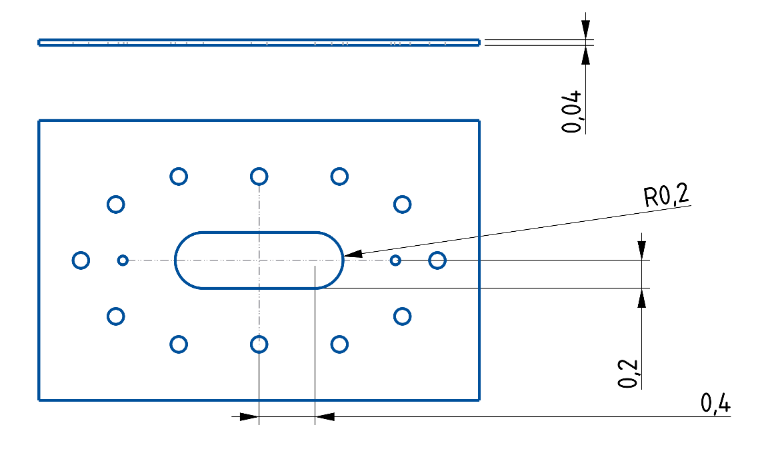


Figure S2: Dimensions of the channel section. (all dimensions are in inches)





Figure S3: Exploded view of various parts. White: Polycarbonate parts. Red: Silicone gaskets. Beige: Polycarbonate membrane (pore size 0.4 µm)


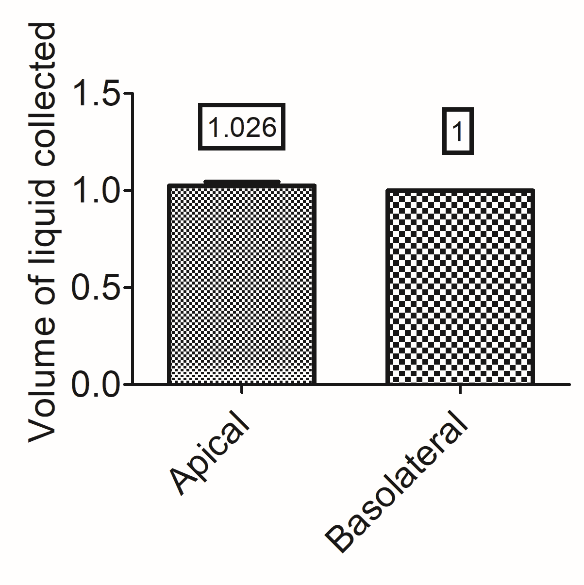


Figure S4: Testing of fluid flow across the porous membrane. No significant difference was observed between the volume collected from the apical (top) and basolateral (bottom) chamber outlets when the inlet flow rate was kept constant.


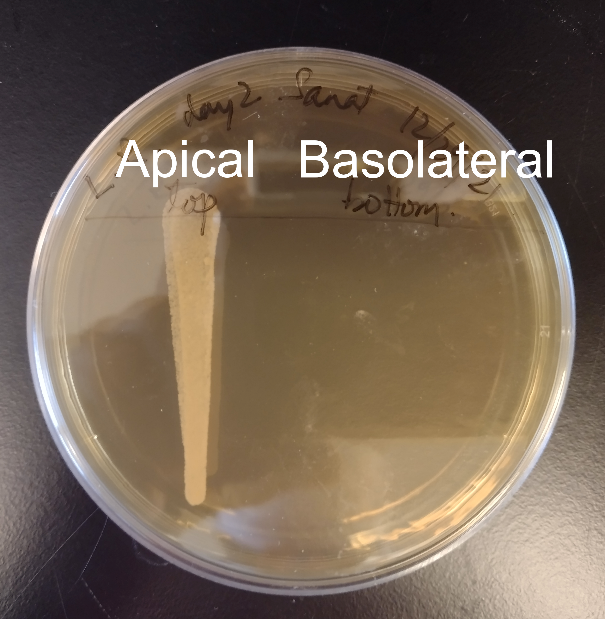


Figure S5: Agar plate where a sample was dropped showing no translocation of *Lactocaseibacillus rhamnosus* (*L. rhamnosus*) to the basolateral chamber 48 hours after the bacteria were seeded into the top chamber of the device

1. (b)


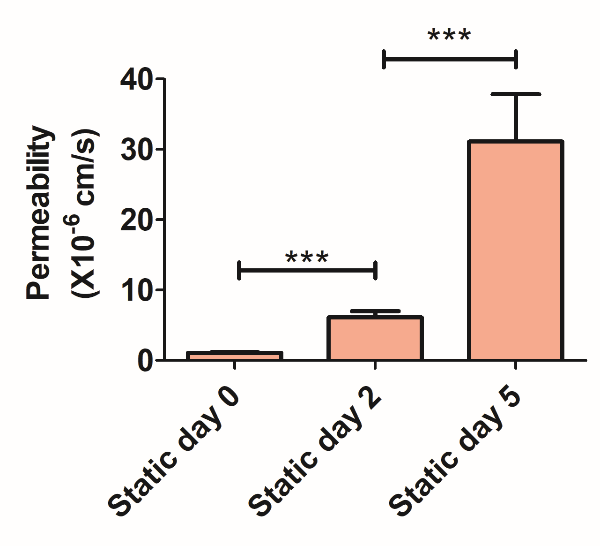

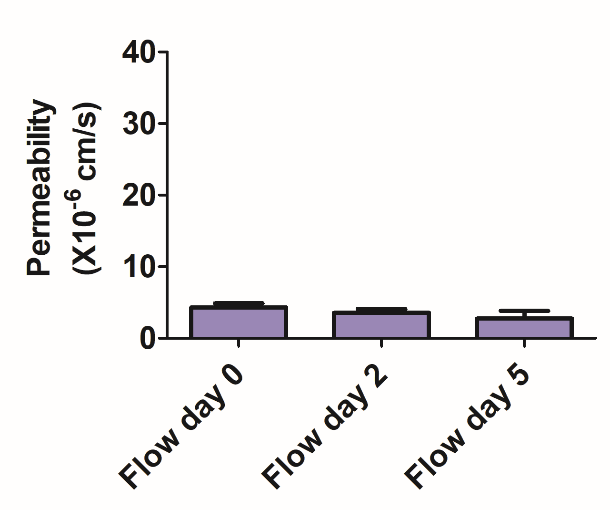


Figure S6: Lucifer yellow permeability assays comparing within (a) static samples and (b) small intestine on a chip (SIOC) samples. Results shown consist of 3 replicates experimental and 2 technical replicates. Error bars indicate standard error of measurement. *** P<0.001 upon comparison using One-way Anova statistical analysis

**2. Supplementary Methods**

**Mucus/MUC2 thickness calculation**

The mucus thickness was quantified using ImageJ software with a user-defined macro based on the fluorescent signals from immunofluorescent images. The fluorescent signals from different channels did not overlap or overlapped minimally, which made the process easier (figure S7). The general algorithm of the process is shown below.

1. Fluorescent images were taken using a laser scanning confocal microscope (LSM 880, Carl Zeiss Microscopy, LLC) with the same settings for replicate samples.
2. Images were imported to ImageJ and the required fluorescent channel was isolated.
3. The fluorescent signals were thresholded (refer to the ImageJ macro below) to encompass only the fluorescent signal region and not the noise. The high and low magnitude of the threshold was kept the same for all replicate images.
4. Volume of the total stained region calculated.
5. Volume was divided by the area of each image to get the average thickness of the stain.


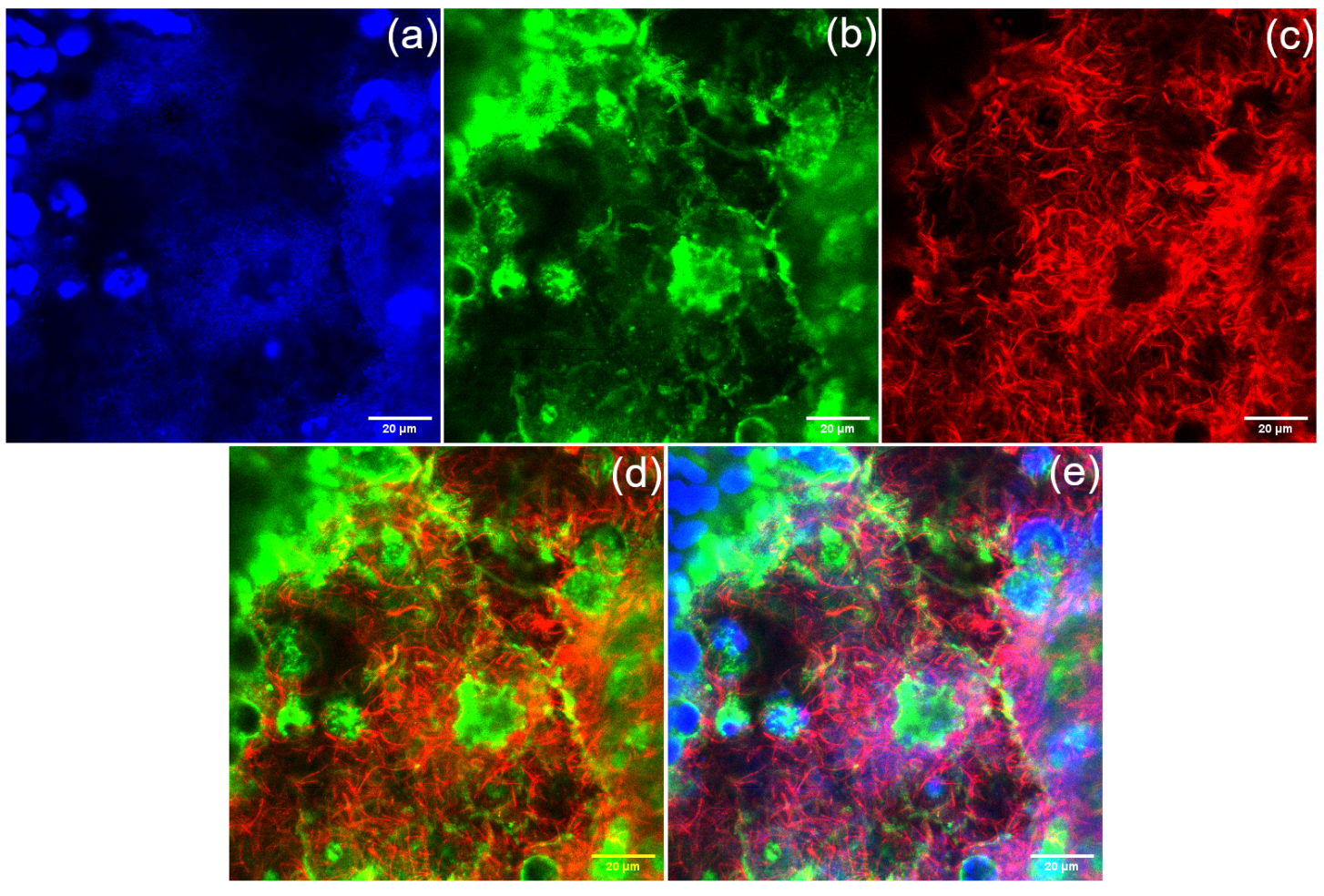


Figure S7: Analysis of a z-stack slice of a sample with *L. rhamnosus* at day 2 of co-culture showing separate localization of mCherry *L. rhamnosus* and wheat germ agglutinin (WGA). (a) Hoechst, (b) mucus- WGA (c) mCherry-*L. rhamnosus*. (d) composite image of WGA and mCherry. (e) composite image of Hoechst, WGA and mCherry.

**ImageJ macro**

// select a directory of images to open

input = getDirectory("input folder where images are stored");

//open a dialogue to select location where results will be stored

output = getDirectory ("output folder for results");

//get list of files in folder

list =getFileList(input);

//setBatchMode(true);

for (j=0; j< list.length; j++){

open(input+list[j]);

//insert the macro here

run ("Split Channels");

close ("C1-*");

//close ("C2-*");

close ("C3-*");

a = newArray();

b = newArray();

for (i=1; i<nSlices+1;i++) {

Stack.setSlice(i);

Stack.getPosition(channel, slice, frame);

//print("Slice number = "+slice);

getStatistics(area,mean);

a[i]= mean;

//print("mean gray value= " +a[i]);

}

for(i=2; i<nSlices+1;i++){

if (a[1] < a[i]){

a[1] = a[i];

c = i;

}

}

//print("the highest intensity is " +a[1]);

//just for confirmation that its going thorugh the sclices

//for (i=1;i<nSlices+1;i++){

//print("mean gray value for slice " + i+ "=" +a[i]);

//}

//print("the highest intensity is " +a[1]+ " which is on slice " +c);

setSlice(c);

setThreshold(18455, 65535, "raw");

run("Clear Results"); // First, clear the results table

// loop through each slice in the stack. Start at n=1 (the first slice),

// keep going while n <= nSlices (nSlices is the total number of slices in the stack)

// and increment n by one after each loop (n++)

for (n=1; n<=nSlices; n++) {

setSlice(n); // set the stack's current slice to n

run("Measure"); // Run the "Measure" function in ImageJ

}

// Create a variable that we will use to store the area measured in each slice

totalArea = 0;

// Loop through each result from 0 (the first result on the table) to nResult (the total number of results on the table)

for (n=0; n < nResults; n++){

totalArea += getResult("Area",n); // Add the area of the current result to the total

}

title = getTitle();

name = getInfo("image.filename");

// Get the calibration information from ImageJ and store into width, height, depth, and unit variables.

// We will only be using depth and unit

getVoxelSize(width, height, depth, unit);

// Calculate the volume by multiplying the sum of area of each slice by the depth

volume = totalArea*depth;

// Print the result of the volume calculation to the log

print(title + " " + volume + " " + unit + "^3");

}
